# Parkinson’s and Alzheimer’s disease impairs temporal precision

**DOI:** 10.21203/rs.3.rs-8628264/v1

**Published:** 2026-01-20

**Authors:** Matthew A. Weber, Christopher M. Hunter, Nandakumar S. Narayanan

**Affiliations:** University of Iowa; University of Iowa; University of Iowa

**Keywords:** Interval timing, Parkinson’s disease, Alzheimer’s disease, Precision, Accuracy

## Abstract

Several studies have reported temporal processing deficits in neurodegenerative diseases such as Parkinson’s and Alzheimer’s disease. These deficits can be quantified by interval timing paradigms that require participants to estimate or produce an interval of several seconds and require working memory for temporal rules as well as attention to time. Timing performance can be quantified by a variety of measures; however, two relatively universal metrics include: 1) temporal accuracy, defined as the mean temporal estimate and 2) temporal precision, reflected by the variability of temporal estimates. We examined temporal accuracy and precision in a meta-analysis of 14 studies in patients with Parkinson’s disease and 10 studies in patients with Alzheimer’s disease. Strikingly, in both diseases, temporal precision was reliably impaired across studies, while temporal accuracy was not. Our meta-analysis suggests that despite the diversity of interval timing paradigms and the complexity of Parkinson’s and Alzheimer’s disease, temporal precision is consistently impaired in these diseases. These results advance interval timing as a reliable assay to study cognitive dysfunction in Parkinson’s and Alzheimer’s disease and may extend to other neurological and psychiatric disorders.

## INTRODUCTION

Interval timing requires participants to estimate a temporal epoch of several seconds by making a motor response and has been studied for more than 150 years (Gibbon, 1977; Gibbon et al., 1984; Staddon, 2005; Vierordt, 1868). While heavily studied in rodent models, human studies suggested that patients with Parkinson’s disease (PD) and Alzheimer’s disease (AD) have impaired interval timing (Nichelli et al., 1993; Pastor et al., 1992). Critically, characterizing timing deficits in PD and AD is important because it could: 1) illuminate neuronal mechanisms in humans (Coull et al., 2011) and how these mechanisms malfunction in neurodegenerative disease; 2) advance translational animal models (Buhusi & Meck, 2005); and 3) add to our existing neuropsychological toolbox.

However, it is unclear how PD and AD affect interval timing, as there have been many competing theories and explanations (Armstrong et al., 2020; Bangert & Balota, 2012; Jones & Jahanshahi, 2014; Meck, 2005; Merchant & De Lafuente, 2014; Q. Zhang et al., 2021). Both diseases have motor features, including slow movements observed in PD (Berardelli et al., 2001) and inaccurate movements observed in AD (Ghilardi et al., 1999). Furthermore, both diseases have marked cognitive deficits that affect executive functions such as working memory and attention (Kensinger et al., 2003) and in other cognitive features, such as declarative memory in AD (Albert, 1996). In addition, more complex deficits in interval timing have been proposed (Malapani et al., 1998, 2002), and temporal deficits in PD have been highly heterogenous (Merchant et al., 2007). Determining how temporal processing deficits relate to universal and quantified metrics such as precision and accuracy would add consistency to measures of interval timing in patients with neurodegenerative disease.

Accordingly, we conducted a meta-analysis of interval timing in PD and AD. From 207 studies with PD and 116 studies with AD, we could extract data from 14 PD and 10 AD studies comparing suprasecond interval timing between a PD/AD population and matched control participants. To our surprise, we found that both PD patients and AD patients had markedly impaired temporal precision but no reliable difference in temporal accuracy, providing insight into timing deficits in PD and AD.

## METHODS

### Search strategy and inclusion/exclusion criteria

An electronic search of PubMed was performed to identify peer-reviewed articles that studied interval timing behavior in patients with Parkinson’s disease (PD) or Alzheimer’s disease (AD). We were interested in two key measures of interval timing behavior: 1) timing accuracy – measure of early or late timing and 2) timing precision – measure of timing variability. Three separate searches were conducted for PD using the search terms “Parkinson’s” and “interval timing” or “time perception” or “time estimation”. We utilized PubMed’s Boolean operator “NOT” to exclude published review articles and Species filter to include only “Human” studies. An identical search was conducted for AD. After removing duplicates, a total of 150 peer-reviewed abstracts for PD and 72 peer-reviewed abstracts for AD remained. Identical searches for both PD and AD were also conducted using PsycINFO. A total of 57 abstracts for PD and 44 abstracts for AD were unique to PsycINFO. All abstracts were then independently screened by two authors (MAW and CH). We synthesized data from studies that measured interval timing behavior using estimation, production, or reproduction tasks above 1 second. Inclusion criteria were: 1) peer-reviewed original research that compared healthy control participants against patients; 2) timed intervals greater than or equal to 1 second; and 3) results reported as timed response or difference in seconds (or milliseconds). Importantly, we required the results to indicate directionality – i.e., a left or right shift in timed response and a decrease or increase in variability. Exclusion criteria were: 1) non-original research, case study, or computational modeling; 2) sub-second durations; 3) duration discrimination tasks (i.e., temporal bisection); 4) temporal order judgement tasks; and 5) motor synchronization tasks. This process resulted in 14 peer-reviewed publications for PD (Honma et al., 2016, 2021; Jones et al., 2008; Y.-C. Kim et al., 2017; Koch et al., 2004, 2008; Perbal et al., 2005; Poryazova et al., 2013; Pouthas & Perbal, 2004; Riesen & Schnider, 2001; Singh et al., 2021; Torta et al., 2010; Wearden et al., 2009; Wojtecki et al., 2011) and 10 peer-reviewed publications for AD (Barabassy et al., 2007; Carrasco et al., 2000; El Haj et al., 2013, 2014; Nichelli et al., 1993; Pai et al., 2021; Ranjbar Pouya et al., 2015; Rueda & Schmitter-Edgecombe, 2009; Sutnikiene et al., 2025; Teghil et al., 2023). From each of these studies, we collected article title, authors, publication year, type of interval timing behavior, number of participants, average accuracy and precision value, and standard deviation (or standard error of the mean; SEM). Most studies directly reported means and standard deviations (or SEMs) in the results section or in table form. However, if these values were not directly reported, we used a plot digitizer tool (Rohatgi, 2019; WebPlotDigitizer: Version 4.2, 2020, https://automeris.io) to extract relevant data.

The goal of this meta-analysis was to include as many data points as possible because of the limited number of manuscripts that met inclusion criteria. As such, there are relevant details to note. Pouthas & Perbal (2004) and Perbal et al. (2005) reported the same accuracy data, which was only extracted from Pouthas & Perbal (2004). Variability data was extracted from Perbal et al. (2005). Kim and colleagues (2017) did not report variability data in the manuscript, so data was obtained from N.S.N and is available with this manuscript. Pai and colleagues (2021) report data from prodromal AD participants. Sutnikiene and colleagues (2025) report data from two AD cohorts: 1) participants that met criteria for mild cognitive impairment due to AD and 2) participants that met criteria for mild dementia and probable AD.

### Statistics

We used R statistical software (v 4.3.2; R Core Team) and the *metafor* meta-analysis package for R (Viechtbauer, 2009, 2010) to calculate standardized mean difference (i.e., Cohen’s *d* effect size) for measures of timing accuracy and timing precision for all studies, meta-regression analyses to determine summary effect sizes, and fixed-effects meta-regression models to determine if either disease has a larger effect on timing precision or accuracy. Importantly, the number of studies reporting the effects of PD or AD on interval timing behavior is limited; thus, we extracted multiple effect sizes when possible, meaning a manuscript may contribute multiple data points from the same participants. Several studies reported multiple interval lengths (10/14 PD; 5/10 AD), different interval timing tasks (6/14 PD; 5/10 AD), or separate cohorts of participants (2/14 PD; 1/10 AD). Finally, effect sizes were adjusted so that a leftward shift in timing or decreased timing variability were reflected by negative values and a rightward shift in timing or increased timing variability were reflected by positive values. Importantly, our meta-analytical approach accounts for multiple effects sizes from the same manuscript or participants by using multivariate/multilevel linear mixed-effects models (*rma.mv* in R). Our statistical approach was checked by Iowa’s Biostatistics and Epidemiology Research and Design Core, and our code and data are available at https://narayanan.lab.uiowa.edu/article/datasets and https://zenodo.org/records/18272471 (M. Weber et al., 2026)

## RESULTS

**Figure 1:**
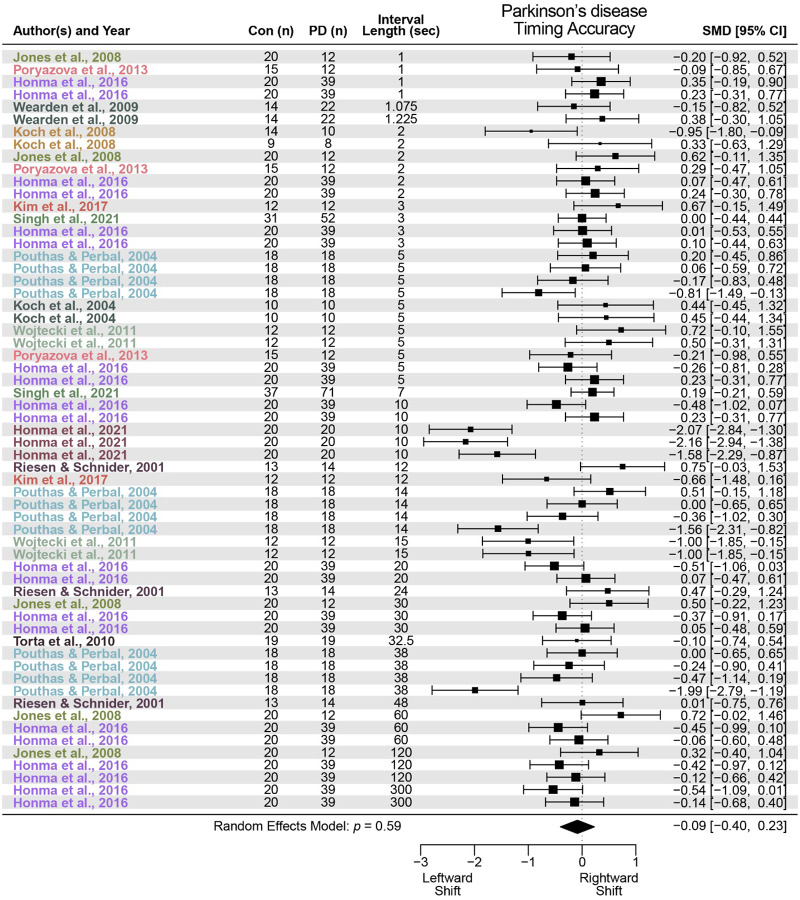
Timing accuracy in Parkinson’s disease (PD). Meta-analysis of 13 prior studies investigating timing accuracy, or measures of early or late timing, in PD patients compared to control participants (Con). PD was not associated with a significant change in timing accuracy (standardized mean difference (SMD) = −0.09; data from 61 effect sizes in 13 studies).

**Figure 2:**
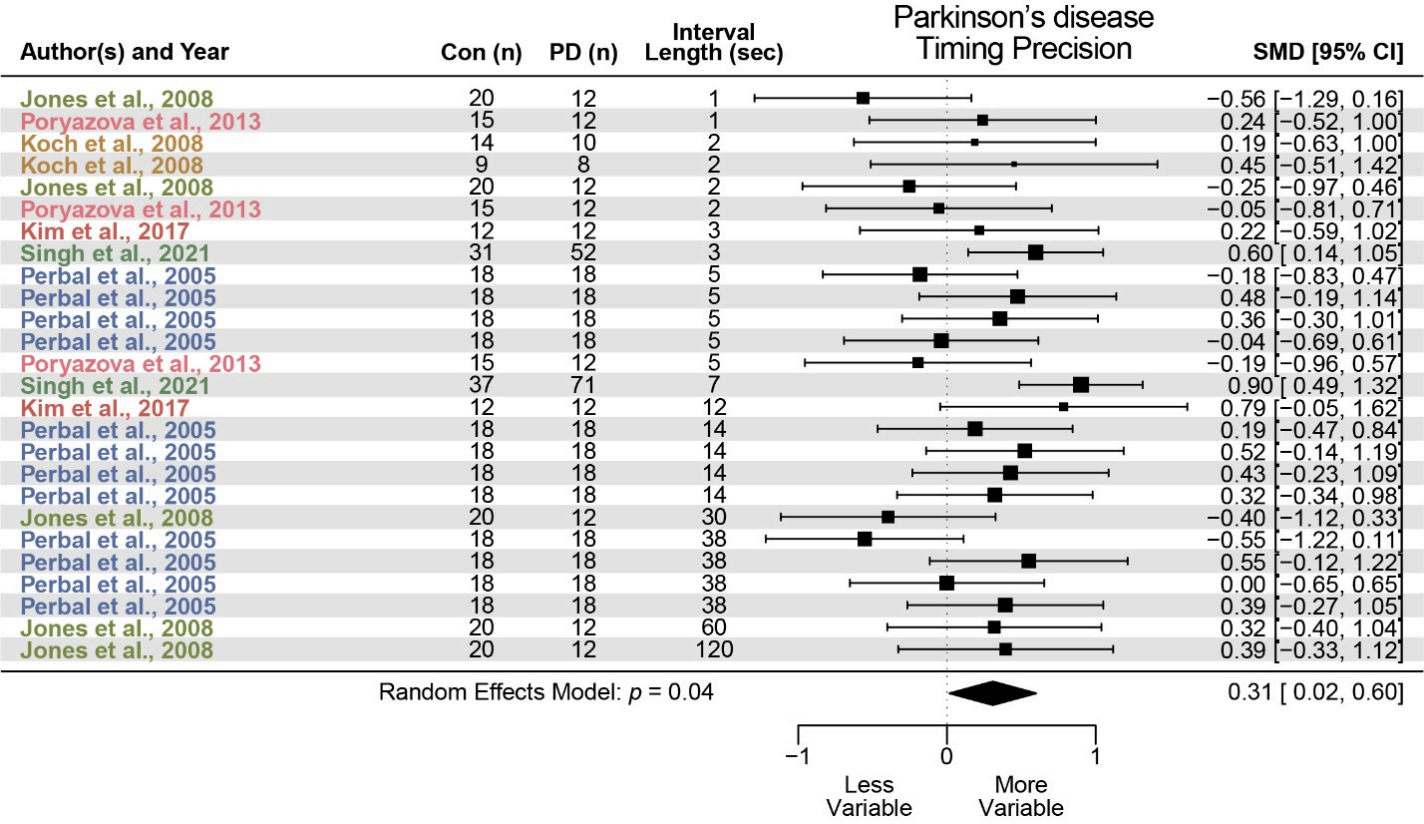
Timing precision in Parkinson’s disease (PD). Meta-analysis of 6 prior studies investigating timing precision, or measures of timing variability, in PD patients compared to control participants (Con). PD was associated with a significant increase in timing variability, or impaired timing precision (standardized mean difference (SMD) = 0.31; data from 26 effect sizes in 6 studies).

We identified 14 studies that met criteria for PD. We found that PD did not significantly alter interval timing accuracy, with a standardized mean difference of −0.09 (95% confidence interval (CI) −0.40–0.23, *p* = 0.59; Fig. 1). However, PD was associated with greater interval timing variability and impaired precision with a standardized mean difference of 0.31 (95% CI 0.02–0.60, *p* = 0.04; Fig. 2). These data indicate no reliable effect of PD on timing accuracy and a small but reliable effect on timing precision.

**Figure 3:**
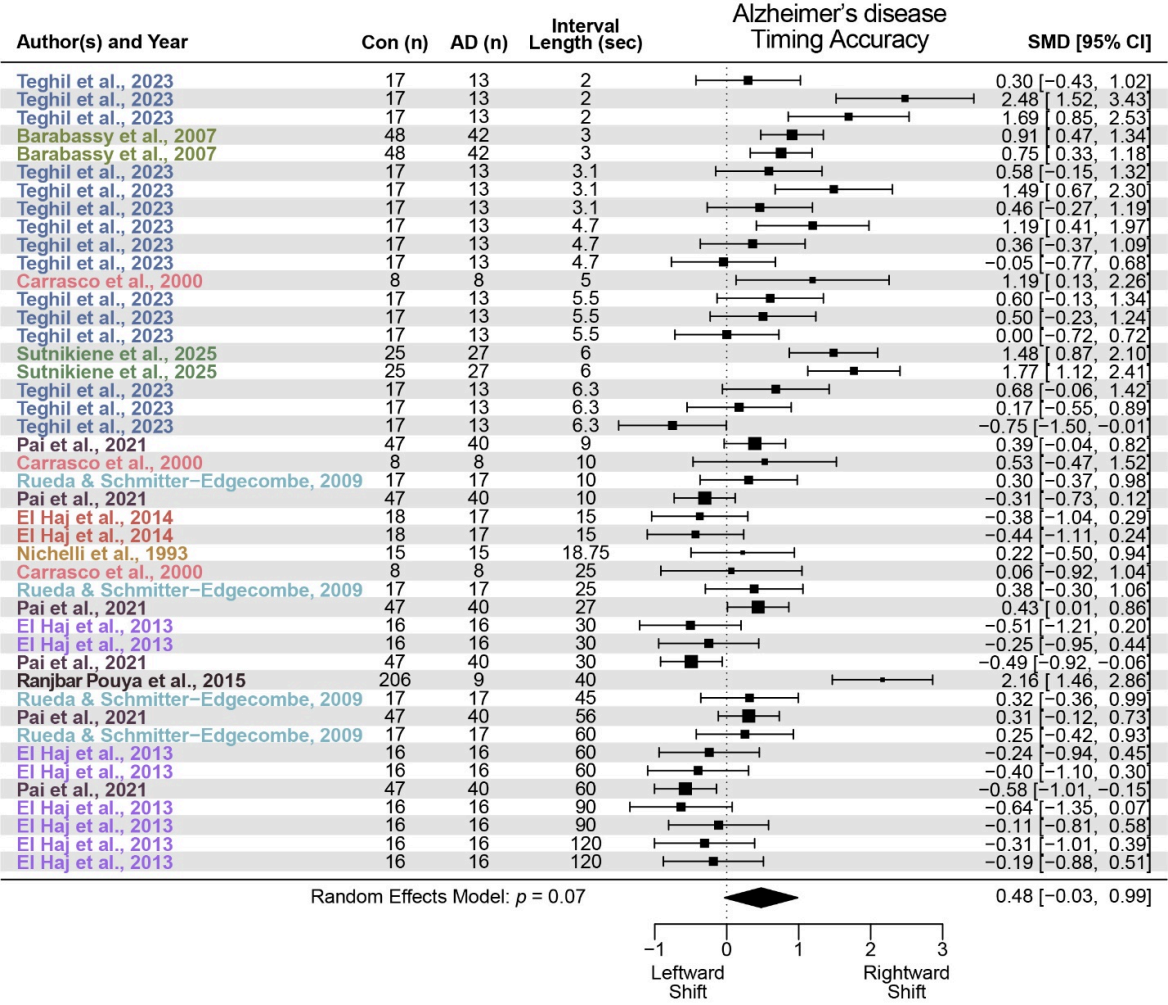
Timing accuracy in Alzheimer’s disease (AD). Meta-analysis of 10 prior studies investigating timing accuracy, or measures of early or late timing, in AD patients compared to control participants (Con). AD was associated with a non-significant rightward shift in timing accuracy (standardized mean difference (SMD) = 0.48; data from 44 effect sizes in 10 studies).

**Figure 4:**
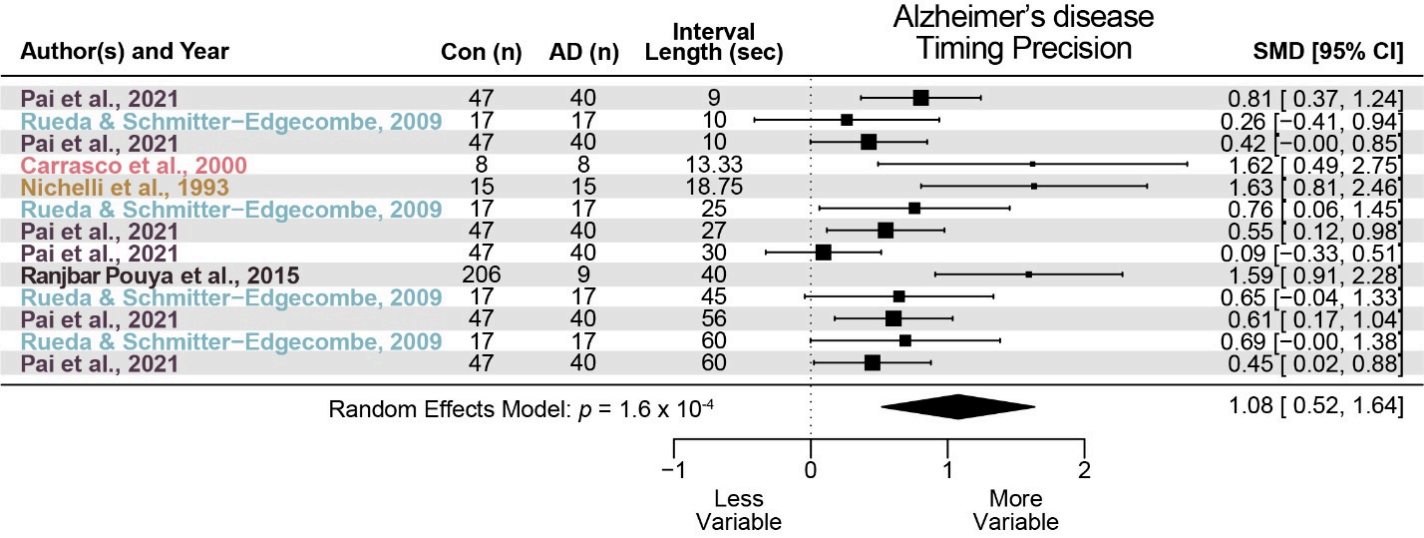
Timing precision in Alzheimer’s disease (AD). Meta-analysis of 5 prior studies investigating timing precision, or measures of timing variability, in AD patients compared to control participants (Con). AD was associated with a significant increase in timing variability, or impaired timing precision (standardized mean difference (SMD) = 1.07; data from 12 effect sizes in 5 studies).

We identified 10 studies that met criteria for AD. Similar to PD, we found that AD did not reliably alter interval timing accuracy with a standardized mean difference of 0.48 (95% CI −0.03–0.99, *p* = 0.07; Fig. 3) but did reliably increase interval timing variability with a standardized mean difference of 1.08 (95% CI 0.52–1.64, *p* = 1.6 × 10^−4^; Fig. 4). These data indicate a moderate but not statistically significant effect of AD on timing accuracy and a large and reliable effect on timing precision.

Finally, a separate meta-regression model indicated that PD had a larger effect on timing variability compared to timing accuracy that did not reach statistical significance (estimate = 0.21, 95% CI −0.01–0.42, z = 1.88, *p* = 0.06). However, a meta-regression model indicated that AD had a significantly larger effect on timing variability compared to timing accuracy (estimate = 0.75, 95% CI 0.37–1.13, z = 3.90, *p* = 9.4 × 10^−5^). Taken together, these data suggest that PD and AD reliably impair temporal precision without affecting temporal accuracy.

## DISCUSSION

We conducted a meta-analysis of interval timing deficits in PD and AD. We focused on studies that used intervals greater than or equal to 1 second from which we could extract statistics on temporal accuracy and precision. We found that both PD and AD reliably affected temporal precision, or the variability of temporal estimates, without affecting temporal accuracy, suggesting that PD and AD increase the variability of interval timing.

Our data suggest that PD and AD decrease temporal precision, regardless of the task specifics. Interestingly, this did not depend on the length of the interval, meaning that perturbations in precision were evident even at intervals as short as a second. Prior work by our group showed that PD patients had impaired temporal precision during a 7-second fixed-interval timing task (Singh et al., 2021; M. A. Weber, Sivakumar, Bova, et al., 2025) and that rodents administered amphetamine had impaired temporal variability (M. A. Weber, Sivakumar, Kirkpatrick, et al., 2025). Of note, impaired temporal variability predicted cognitive dysfunction as measured by the Montreal Cognitive Assessment (Dalrymple-Alford et al., 2010; Nasreddine et al., 2005; Singh et al., 2021). However, cognitive function and other patient-specific factors were not uniformly measured across included studies, so we are unable to determine which aspects of PD or AD best predict temporal variability. Future studies will determine how specific neuropsychological metrics and measures of brain activity best account for impaired temporal precision in PD or AD.

PD has been traditionally considered a movement disorder (Berardelli et al., 2001). On reaction-time and other motor-specific tasks, PD patients are often slow, consistent with the more generalized bradykinesia in PD (Evarts et al., 1981; Singh et al., 2023). Further, AD certainly has motor features, such as slowed and inaccurate movements (Ghilardi et al., 1999). However, these features cannot easily explain increased temporal variability, as slow movements would result in decreased temporal accuracy without changes in temporal precision. Rather, there may be deficits in executive processing, such as impaired working memory to maintain temporal rules or inattention to the passage of time, that is degrading temporal precision by increasing temporal variability. Future studies will explicitly measure executive function and link these measures to interval timing accuracy and precision.

PD has been better studied in interval timing, leading to theoretical advances on the nature of timing deficits. These deficits have been heterogenous (Merchant et al., 2007); however both PD and AD are complex diseases with many differences in patient-specific factors within each disease category. Some studies have implicated impulsivity (J. Zhang et al., 2016) or motor or perceptual factors (Jones & Jahanshahi, 2014). Other investigators have implicated basal ganglia mechanisms (Malapani et al., 1998), although both PD and AD involve broad brain networks including cortical and subcortical networks (S. Kim et al., 2022; Parker et al., 2013). Our work is important because despite this complexity, both PD and AD patients have decreased temporal precision in interval timing with no reliable effect on temporal accuracy. This finding is helpful in distilling temporal deficits in PD and AD to a single measurable parameter.

Meta-analyses such as ours are inherently limited. First, we could not include every study because they did not meet our inclusion criteria, lacked a control group, or met exclusion criteria. Second, there were myriad nuances between studies that could not be fully captured in a meta-analysis. Third, PD and AD are diseases with distinct pathological processes. While we hypothesize that impairments in frontal brain structures underlie impairments in temporal precision, future work is needed to rigorously test this idea.

In summary, we find that PD and AD patients have decreased temporal precision without reliable effects on temporal accuracy. Our findings capture disruptions in temporal processing in neurodegenerative disease, which will advance new assays and inspire new investigations into their neural substrates.

## Data Availability

All data and code are available at https://narayanan.lab.uiowa.edu/article/datasets and https://zenodo.org/records/18272471.

## References

[R1] Albert M. S. (1996). Cognitive and neurobiologic markers of early Alzheimer disease. Proceedings of the National Academy of Sciences of the United States of America, 93(24), 13547–13551. 10.1073/pnas.93.24.13547 8942970 PMC33644

[R2] Armstrong P., Pardon M.-C., & Bonardi C. (2020). Timing impairments in early Alzheimer’s disease: Evidence from a mouse model. Behavioral Neuroscience, 134(2), 82–100. 10.1037/bne0000359 32175759

[R3] Bangert A. S., & Balota D. A. (2012). Keep Up the Pace: Declines in Simple Repetitive Timing Differentiate Healthy Aging from the Earliest Stages of Alzheimer’s Disease. Journal of the International Neuropsychological Society, 18(6), 1052–1063. 10.1017/S1355617712000860 22929329 PMC3505757

[R4] Barabassy A., Beinhoff U., & Riepe M. W. (2007). Cognitive estimation in mild Alzheimer’s disease. Journal of Neural Transmission (Vienna, Austria: 1996), 114(11), 1479–1484. 10.1007/s00702-007-0752-2 17520318

[R5] Berardelli A., Rothwell J. C., Thompson P. D., & Hallett M. (2001). Pathophysiology of bradykinesia in Parkinson’s disease. Brain: A Journal of Neurology, 124(Pt 11), 2131–2146. 10.1093/brain/124.11.2131 11673316

[R6] Buhusi C. V., & Meck W. H. (2005). What makes us tick? Functional and neural mechanisms of interval timing. Nature Reviews Neuroscience, 6(10), 755–765. 10.1038/nrn1764 16163383

[R7] Carrasco M. C., Guillem M. J., & Redolat R. (2000). Estimation of short temporal intervals in Alzheimer’s disease. Experimental Aging Research, 26(2), 139–151. 10.1080/036107300243605 10755220

[R8] Coull J. T., Cheng R.-K., & Meck W. H. (2011). Neuroanatomical and Neurochemical Substrates of Timing. Neuropsychopharmacology, 36(1), 3–25. 10.1038/npp.2010.113 20668434 PMC3055517

[R9] Dalrymple-Alford J. C., MacAskill M. R., Nakas C. T., Livingston L., Graham C., Crucian G. P., Melzer T. R., Kirwan J., Keenan R., Wells S., Porter R. J., Watts R., & Anderson T. J. (2010). The MoCA: Well-suited screen for cognitive impairment in Parkinson disease. Neurology, 75(19), 1717–1725. 10.1212/WNL.0b013e3181fc29c9 21060094

[R10] El Haj M., Moroni C., Samson S., Fasotti L., & Allain P. (2013). Prospective and retrospective time perception are related to mental time travel: Evidence from Alzheimer’s disease. Brain and Cognition, 83(1), 45–51. 10.1016/j.bandc.2013.06.008 23872099

[R11] El Haj M., Omigie D., & Moroni C. (2014). Time reproduction during high and low attentional tasks in Alzheimer’s Disease. “A watched kettle never boils.” Brain and Cognition, 88, 1–5. 10.1016/j.bandc.2014.04.002 24794142

[R12] Evarts E. V., Teräväinen H., & Calne D. B. (1981). Reaction time in Parkinson’s disease. Brain: A Journal of Neurology, 104(Pt 1), 167–186. 10.1093/brain/104.1.167 7470841

[R13] Ghilardi M. F., Alberoni M., Marelli S., Rossi M., Franceschi M., Ghez C., & Fazio F. (1999). Impaired movement control in Alzheimer’s disease. Neuroscience Letters, 260(1), 45–48. 10.1016/s0304-3940(98)00957-4 10027696

[R14] Gibbon J. (1977). Scalar expectancy theory and Weber’s law in animal timing. Psychological Review, 84(3), 279–325. 10.1037/0033-295X.84.3.279

[R15] Gibbon J., Church R. M., & Meck W. H. (1984). Scalar timing in memory. Annals of the New York Academy of Sciences, 423, 52–77. 10.1111/j.1749-6632.1984.tb23417.x 6588812

[R16] Honma M., Kuroda T., Futamura A., Shiromaru A., & Kawamura M. (2016). Dysfunctional counting of mental time in Parkinson’s disease. Scientific Reports, 6, 25421. 10.1038/srep25421 27146904 PMC4857080

[R17] Honma M., Murakami H., Yabe Y., Kuroda T., Futamura A., Sugimoto A., Terao Y., Masaoka Y., Izumizaki M., Kawamura M., & Ono K. (2021). Stopwatch training improves cognitive functions in patients with Parkinson’s disease. Journal of Neuroscience Research, 99(5), 1325–1336. 10.1002/jnr.24812 33594677

[R18] Jones C. R. G., & Jahanshahi M. (2014). Motor and Perceptual Timing in Parkinson’s Disease. In Merchant H. & De Lafuente V. (Eds.), Neurobiology of Interval Timing (Vol. 829, pp. 265–290). Springer New York. 10.1007/978-1-4939-1782-2_14 25358715

[R19] Jones C. R. G., Malone T. J. L., Dirnberger G., Edwards M., & Jahanshahi M. (2008). Basal ganglia, dopamine and temporal processing: Performance on three timing tasks on and off medication in Parkinson’s disease. Brain and Cognition, 68(1), 30–41. 10.1016/j.bandc.2008.02.121 18378374

[R20] Kensinger E. A., Shearer D. K., Locascio J. J., Growdon J. H., & Corkin S. (2003). Working memory in mild Alzheimer’s disease and early Parkinson’s disease. Neuropsychology, 17(2), 230–239. 10.1037/0894-4105.17.2.230 12803428

[R21] Kim S., Nam Y., Kim H. S., Jung H., Jeon S. G., Hong S. B., & Moon M. (2022). Alteration of Neural Pathways and Its Implications in Alzheimer’s Disease. Biomedicines, 10(4), 845. 10.3390/biomedicines10040845 35453595 PMC9025507

[R22] Kim Y.-C., Han S.-W., Alberico S. L., Ruggiero R. N., De Corte B., Chen K.-H., & Narayanan N. S. (2017). Optogenetic Stimulation of Frontal D1 Neurons Compensates for Impaired Temporal Control of Action in Dopamine-Depleted Mice. Current Biology, 27(1), 39–47. 10.1016/j.cub.2016.11.029 27989675 PMC5225083

[R23] Koch G., Costa A., Brusa L., Peppe A., Gatto I., Torriero S., Gerfo E. L., Salerno S., Oliveri M., Carlesimo G. A., & Caltagirone C. (2008). Impaired reproduction of second but not millisecond time intervals in Parkinson’s disease. Neuropsychologia, 46(5), 1305–1313. 10.1016/j.neuropsychologia.2007.12.005 18215403

[R24] Koch G., Oliveri M., Brusa L., Stanzione P., Torriero S., & Caltagirone C. (2004). High-frequency rTMS improves time perception in Parkinson disease. Neurology, 63(12), 2405–2406. 10.1212/01.wnl.0000147336.19972.82 15623713

[R25] Malapani C., Deweer B., & Gibbon J. (2002). Separating storage from retrieval dysfunction of temporal memory in Parkinson’s disease. Journal of Cognitive Neuroscience, 14(2), 311–322. 10.1162/089892902317236920 11970794

[R26] Malapani C., Rakitin B., Levy R., Meck W. H., Deweer B., Dubois B., & Gibbon J. (1998). Coupled Temporal Memories in Parkinson’s Disease: A Dopamine-Related Dysfunction. Journal of Cognitive Neuroscience, 10(3), 316–331. 10.1162/089892998562762 9869707

[R27] Meck W. H. (2005). Neuropsychology of timing and time perception. Brain and Cognition, 58(1), 1–8. 10.1016/j.bandc.2004.09.004 15878722

[R28] Merchant H., & De Lafuente V. (2014). Introduction to the Neurobiology of Interval Timing. In Merchant H. & De Lafuente V. (Eds.), Neurobiology of Interval Timing (Vol. 829, pp. 1–13). Springer New York. 10.1007/978-1-4939-1782-2_1 25358702

[R29] Merchant H., Luciana M., Hooper C., Majestic S., & Tuite P. (2007). Interval timing and Parkinson’s disease: Heterogeneity in temporal performance. Experimental Brain Research, 184(2), 233–248. 10.1007/s00221-007-1097-7 17828600

[R30] Nasreddine Z. S., Phillips N. A., Bédirian V., Charbonneau S., Whitehead V., Collin I., Cummings J. L., & Chertkow H. (2005). The Montreal Cognitive Assessment, MoCA: A brief screening tool for mild cognitive impairment. Journal of the American Geriatrics Society, 53(4), 695–699. 10.1111/j.1532-5415.2005.53221.x 15817019

[R31] Nichelli P., Venneri A., Molinari M., Tavani F., & Grafman J. (1993). Precision and accuracy of subjective time estimation in different memory disorders. Brain Research. Cognitive Brain Research, 1(2), 87–93. 10.1016/0926-6410(93)90014-v 8513243

[R32] Pai M.-C., Yang C.-J., & Fan S.-Y. (2021). Time Perception in Prodromal Alzheimer’s Dementia and in Prodromal Dementia With Lewy Bodies. Frontiers in Psychiatry, 12, 728344. 10.3389/fpsyt.2021.728344 34690834 PMC8529046

[R33] Parker K. L., Lamichhane D., Caetano M. S., & Narayanan N. S. (2013). Executive dysfunction in Parkinson’s disease and timing deficits. Frontiers in Integrative Neuroscience, 7. 10.3389/fnint.2013.00075 PMC3813949 24198770

[R34] Pastor M. A., Artieda J., Jahanshahi M., & Obeso J. A. (1992). Time estimation and reproduction is abnormal in Parkinson’s disease. Brain: A Journal of Neurology, 115 Pt 1, 211–225. 10.1093/brain/115.1.211 1559155

[R35] Perbal S., Deweer B., Pillon B., Vidailhet M., Dubois B., & Pouthas V. (2005). Effects of internal clock and memory disorders on duration reproductions and duration productions in patients with Parkinson’s disease. Brain and Cognition, 58(1), 35–48. 10.1016/j.bandc.2005.02.003 15878725

[R36] Poryazova R., Mensen A., Bislimi F., Huegli G., Baumann C. R., & Khatami R. (2013). Time perception in narcolepsy in comparison to patients with Parkinson’s disease and healthy controls—An exploratory study. Journal of Sleep Research, 22(6), 625–633. 10.1111/jsr.12069 23879404

[R37] Pouthas V., & Perbal S. (2004). Time perception depends on accurate clock mechanisms as well as unimpaired attention and memory processes. Acta Neurobiologiae Experimentalis, 64(3), 367–385. 10.55782/ane-2004-1520 15283479

[R38] Ranjbar Pouya O., Kelly D. M., & Moussavi Z. (2015). Tendency to overestimate the explicit time interval in relation to aging and cognitive decline. Annual International Conference of the IEEE Engineering in Medicine and Biology Society. IEEE Engineering in Medicine and Biology Society. Annual International Conference, 2015, 4692–4695. 10.1109/EMBC.2015.7319441 26737341

[R39] Riesen J. M., & Schnider A. (2001). Time estimation in Parkinson’s disease: Normal long duration estimation despite impaired short duration discrimination. Journal of Neurology, 248(1), 27–35. 10.1007/s004150170266 11266017

[R40] Rohatgi A. (2019). WebPoltDigitizer (Version 4.4) [Computer software].

[R41] Rueda A. D., & Schmitter-Edgecombe M. (2009). Time estimation abilities in mild cognitive impairment and Alzheimer’s disease. Neuropsychology, 23(2), 178–188. 10.1037/a0014289 19254091

[R42] Singh A., Cole R. C., Espinoza A. I., Evans A., Cao S., Cavanagh J. F., & Narayanan N. S. (2021). Timing variability and midfrontal ~4 Hz rhythms correlate with cognition in Parkinson’s disease. Npj Parkinson’s Disease, 7(1), 14. 10.1038/s41531-021-00158-x PMC7884691 33589640

[R43] Singh A., Cole R. C., Espinoza A. I., Wessel J. R., Cavanagh J. F., & Narayanan N. S. (2023). Evoked midfrontal activity predicts cognitive dysfunction in Parkinson’s disease. Journal of Neurology, Neurosurgery & Psychiatry, 94(11), 945–953. 10.1136/jnnp-2022-330154 37263767 PMC10592174

[R44] Staddon J. (2005). Interval timing: Memory, not a clock. Trends in Cognitive Sciences, 9(7), 312–314. 10.1016/j.tics.2005.05.013 15953755

[R45] Sutnikiene V., Pakulaite-Kazliene G., Audronyte E., Kuzmickaite J., & Kaubrys G. (2025). Time/Movement Estimation and Mental Rotation Tasks as Early Cognitive Markers in Alzheimer’s Disease. Brain and Behavior, 15(11), e71077. 10.1002/brb3.71077 41261930 PMC12631017

[R46] Teghil A., Boccia M., Di Vita A., Zazzaro G., Sepe Monti M., Trebbastoni A., Talarico G., Campanelli A., Bruno G., Guariglia C., de Lena C., & D’Antonio F. (2023). Multidimensional assessment of time perception along the continuum of Alzheimer’s Disease and evidence of alterations in subjective cognitive decline. Scientific Reports, 13(1), 22117. 10.1038/s41598-023-49222-x 38092802 PMC10719320

[R47] Torta D. M. E., Castelli L., Latini-Corazzini L., Banche A., Lopiano L., & Geminiani G. (2010). Dissociation between time reproduction of actions and of intervals in patients with Parkinson’s disease. Journal of Neurology, 257(8), 1356–1361. 10.1007/s00415-010-5532-5 20352253

[R48] Viechtbauer W. (2009). metafor: Meta-Analysis Package for R (p. 4.6–0) [Dataset]. 10.32614/CRAN.package.metafor

[R49] Viechtbauer W. (2010). Conducting Meta-Analyses in *R* with the metafor Package. Journal of Statistical Software, 36(3). 10.18637/jss.v036.i03

[R50] Vierordt K. (1868). Der zeitsinn nach versuchen.

[R51] Wearden J. H., Smith-Spark J. H., Cousins R., Edelstyn N. M. J., Cody F. W. J., & O’Boyle D. J. (2009). Effect of click trains on duration estimates by people with Parkinson’s disease. Quarterly Journal of Experimental Psychology (2006), 62(1), 33–40. 10.1080/17470210802229047 18622809

[R52] Weber M. A., Sivakumar K., Bova A. S., Tabakovic E. E., Conlon M. M., Oya M., Cole R. C., Espinoza A. I., Kim Y., & Narayanan N. S. (2025). Ventral tegmental area dopamine controls timing variability. Neuroscience. 10.1101/2025.10.27.684913

[R53] Weber M. A., Sivakumar K., Kirkpatrick B. Q., Stutt H. R., Tabakovic E. E., Bova A. S., Kim Y.-C., & Narayanan N. S. (2025). Amphetamine increases timing variability by degrading prefrontal temporal encoding. Neuropharmacology, 275, 110486. 10.1016/j.neuropharm.2025.110486 40324651 PMC12261982

[R54] Weber M., Hunter C., & Narayanan N. (2026). Data for Parkinson’s and Alzheimer’s disease impairs temporal precision [Dataset]. Zenodo. 10.5281/ZENODO.18272470

[R55] Wojtecki L., Elben S., Timmermann L., Reck C., Maarouf M., Jörgens S., Ploner M., Südmeyer M., Groiss S. J., Sturm V., Niedeggen M., & Schnitzler A. (2011). Modulation of human time processing by subthalamic deep brain stimulation. PloS One, 6(9), e24589. 10.1371/journal.pone.0024589 21931767 PMC3171456

[R56] Zhang J., Nombela C., Wolpe N., Barker R. A., & Rowe J. B. (2016). Time on timing: Dissociating premature responding from interval sensitivity in Parkinson’s disease. Movement Disorders, 31(8), 1163–1172. 10.1002/mds.26631 27091513 PMC4988382

[R57] Zhang Q., Weber M. A., & Narayanan N. S. (2021). Medial prefrontal cortex and the temporal control of action. International Review of Neurobiology, 158, 421–441. 10.1016/bs.irn.2020.11.004 33785154 PMC8875599

